# Supplementary material for: Resolving the full spectrum of human genome variation using Linked-Reads
Source: Genome Res. 2019 Apr;29(4):635–45. doi: 10.1101/gr.234443.118 (PMC6442396; doi:10.1101/gr.234443.118)
Supplement: Supplemental Material [file supp_gr.234443.118_Supplemental_longranger-2.2.zip › longranger-2.2.tar.bz2!/lr2.2-public/lib/stan/cmdstan/stan/doc/api/html/check__ldlt__factor_8hpp_source.html]

Stan: src/stan/math/error\_handling/matrix/check\_ldlt\_factor.hpp Source File


|  |  |
| --- | --- |
|  | Stan  2.5.0  probability, sampling & optimization |


- Main Page
- Related Pages
- Namespaces
- Classes
- Files

- File List
- File Members

All Classes Namespaces Files Functions Variables Typedefs Enumerations Enumerator Friends Macros Pages

- src
- stan
- math
- error\_handling
- matrix

check\_ldlt\_factor.hpp

Go to the documentation of this file.

1 #ifndef STAN\_\_MATH\_\_ERROR\_HANDLING\_\_MATRIX\_\_CHECK\_LDLT\_FACTOR\_HPP

2 #define STAN\_\_MATH\_\_ERROR\_HANDLING\_\_MATRIX\_\_CHECK\_LDLT\_FACTOR\_HPP

3

4 #include <sstream>

5 #include <stan/math/matrix/Eigen.hpp>

6 #include <stan/math/error\_handling/dom\_err.hpp>

7 #include <stan/math/matrix/LDLT\_factor.hpp>

8

9 namespace stan {

10  namespace math {

11

23  template <typename T, int R, int C, typename T\_result>

24  inline bool check\_ldlt\_factor(const char\* function,

25  stan::math::LDLT\_factor<T,R,C> &A,

26  const char\* name,

27  T\_result\* result) {

28  if (!A.success()) {

29  std::ostringstream message;

30  message << "underlying matrix is not positive definite. "

31  << name << "last conditional variance is %1%.";

32  std::string msg(message.str());

33  const T\_result too\_small = A.vectorD().tail(1)(0);

34  return dom\_err(function,too\_small,name,msg.c\_str(),"",result);

35  }

36  return true;

37  }

38

39  }

40 }

41 #endif

dom\_err.hpp

stan::math::LDLT\_factor< T, R, C >::vectorD

Eigen::Matrix< T, Eigen::Dynamic, 1 > vectorD() const

**Definition:** LDLT\_factor.hpp:100

stan::math::dom\_err

bool dom\_err(const char \*function, const T &y, const char \*name, const char \*error\_msg, const T\_msg error\_msg2, T\_result \*result)

**Definition:** dom\_err.hpp:36

stan::math::check\_ldlt\_factor

bool check\_ldlt\_factor(const char \*function, stan::math::LDLT\_factor< T, R, C > &A, const char \*name, T\_result \*result)

Return true if the underlying matrix is positive definite.

**Definition:** check\_ldlt\_factor.hpp:24

stan::math::LDLT\_factor< T, R, C >::success

bool success() const

**Definition:** LDLT\_factor.hpp:73

Eigen.hpp

stan::math::LDLT\_factor< T, R, C >

LDLT\_factor is a thin wrapper on Eigen::LDLT to allow for reusing factorizations and efficient autodi...

**Definition:** LDLT\_factor.hpp:56

LDLT\_factor.hpp


---

[ Stan Home Page ]

*© 2011–2014,
Stan Development Team.*
